# Genetic Polymorphisms in Monoamine Systems and Outcome of Cognitive Behavior Therapy for Social Anxiety Disorder

**DOI:** 10.1371/journal.pone.0079015

**Published:** 2013-11-15

**Authors:** Evelyn Andersson, Christian Rück, Catharina Lavebratt, Erik Hedman, Martin Schalling, Nils Lindefors, Elias Eriksson, Per Carlbring, Gerhard Andersson, Tomas Furmark

**Affiliations:** 1 Department of Clinical Neuroscience, Division of Psychiatry, Karolinska Institutet, Stockholm, Sweden; 2 Department of Molecular Medicine, Center for Molecular Medicine Neurogenetics Unit, Karolinska Institutet, Stockholm, Sweden; 3 Department of Clinical Neuroscience, Osher Center for Integrative Medicine & Division of Psychology, Karolinska Institutet, Stockholm, Sweden; 4 Department of Pharmacology, Institute of Neuroscience, Sahlgrenska Academy, University of Gothenburg, Gothenburg, Sweden; 5 Department of Psychology, Stockholm University, Stockholm, Sweden; 6 Department of Behavior Sciences and Learning, Swedish Institute for Disability Research, Linköping University, Linköping, Sweden; 7 Department of Psychology, Uppsala University, Uppsala, Sweden; University of Wuerzburg, Germany

## Abstract

**Objective:**

The role of genetics for predicting the response to cognitive behavior therapy (CBT) for social anxiety disorder (SAD) has only been studied in one previous investigation. The *serotonin transporter* (*5-HTT*LPR), the *catechol-o-methyltransferase* (*COMT*) val158met, and the *tryptophan hydroxylase-2* (*TPH2*) G-703Tpolymorphisms are implicated in the regulation of amygdala reactivity and fear extinction and therefore might be of relevance for CBT outcome. The aim of the present study was to investigate if these three gene variants predicted response to CBT in a large sample of SAD patients.

**Method:**

Participants were recruited from two separate randomized controlled CBT trials (trial 1: n = 112, trial 2: n = 202). Genotyping were performed on DNA extracted from blood or saliva samples. Effects were analyzed at follow-up (6 or 12 months after treatment) for both groups and for each group separately at post-treatment. The main outcome measure was the Liebowitz Social Anxiety Scale Self-Report.

**Results:**

At long-term follow-up, there was no effect of any genotype, or gene × gene interactions, on treatment response. In the subsamples, there was time by genotype interaction effects indicating an influence of the *TPH2* G-703T-polymorphism on CBT short-term response, however the direction of the effect was not consistent across trials.

**Conclusions:**

None of the three gene variants, *5-HTT*LPR, *COMT*val158met and *TPH2* G-703T, was associated with long-term response to CBT for SAD.

**Trial Registration:**

ClinicalTrials.gov (ID-NCT0056496)

## Introduction

Even though cognitive behavior therapy (CBT) is an effective treatment of anxiety disorders, many patients (25–50%) do not respond sufficiently [1], [2]. Therefore, there is a need to improve not only the treatments but also how patients are selected for treatment in order to optimize the efficacy [3], [4]. Until recently, the focus of genetic studies has been limited to addressing the etiology of anxiety disorders rather than the outcome of psychological treatment. However, the expanding field of therapygenetics attempts to explore the relationship between genetic variation and psychological treatment response [5]–[7]. Ultimately, such knowledge could be used to tailor therapies based on patients' biological markers, which in turn, could improve therapeutic outcome.

In patients with anxiety disorders, neural activity in the amygdala is reported to be predictive of both pharmacological [8] and psychosocial treatment outcomes including CBT [9], [10]. Fear extinction is the process when patients approach the feared stimuli in a prolonged, repeated, and gradual manner until the conditioned fear response subsides. This is a crucial element of exposure-based therapy and is thought to be an active safety learning process leading to chemical and structural changes in the brain's synaptic processes [11] which might be of relevance for the anxiolytic outcome of CBT. Monoamine-related gene polymorphisms have previously been tied to amygdala reactivity [10], [12], treatment efficacy [5], [13]–[15] and fear extinction processes [16], [17] and might therefore influence the outcome of CBT.

The *serotonin transporter* (*5-HTT*) linked polymorphic region (*5-HTT*LPR), a 43-bp insertion/deletion located in the promoter of *5-HTT*, renders a long (l) and a short (s) allele. The s-allele of *5-HTT*LPR is associated with reduction of serotonin uptake and 5-HTT expression [18]. Having the s-allele is associated with increased amygdala reactivity [10], [19]–[21] and poorer treatment response to selective serotonin reuptake inhibitors (SSRIs) for social anxiety disorder (SAD) [22] and panic disorder [23]. In another study persistent higher symptoms in panic disorder patients carrying either an s-allele or an L_G_-allele, were reported by Lonsdorf et al. [24]. In line with this, one study reported that carrying the s-allele predicted poorer response to CBT for post traumatic stress disorder [25]. Furthermore, higher resistance to fear extinction has been demonstrated in s-carriers [16]. However, in a study on children with anxiety, ss homozygosity was associated with better outcome of CBT [5] suggesting that the s-allele might be a high-responsive allele rather than a specific risk-allele [26]. Yet, a small-scale study of SAD found no effect of *5-HTT*LPR on CBT outcome [3].

Cathecol-o-methyltransferase (COMT) degrades released extracellular dopamine, and the gene has a functional single nucleotide polymorphism (SNP) in exon 3 at codon 158, called *COMT*val158met. The met (methionine)-allele is associated with lower enzyme activity, and reduces the effectiveness of dopamine degradation and thereby increases the synaptic availability of dopamine [27]. This has in turn been suggested to affect fear extinction via fear memory consolidation [28]. Neuroimaging studies have shown association between the *COMT*val158met polymorphism and amygdala reactivity, although not in a uniform way [29]. In another study, adult patients with panic disorder, and the metmet genotype profited less from CBT than patients carrying at least one val-allele, however, only an association with outcome of the exposure-based parts of the treatment was observed, but not for the cognitive restructuring parts[13]. Consistently, Lonsdorf and colleagues [30] also showed compromised fear extinction in metmet carriers. This was not replicated in a trial by Ågren and colleagues [16], however, the two studies differed somewhat in design where the latter used a reconsolidation- or memory reactivation protocol before extinction, something that potentially could contribute to the discrepancy of findings between the two studies.

Finally, tryptophan hydroxylase-2 (TPH2) is involved in the rate-limiting process in the first steps of serotonin synthesis. *TPH2* holds a polymorphism, G-703T, in the promoter region where the T-variant of is associated with amygdala hyper responsivity both in healthy individuals [12], [31] and in patients with SAD [10], [32]. Moreover, SAD patients carrying T alleles had poorer placebo response when treated under randomized double-blinded conditions [10]. The *TPH2* G-703T is also associated with fear conditioning processes [33]. In a recent study of pharmacological treatment of depression and anxiety among children and adolescents, the authors reported an additive effect of the *5-HTT*LPR s-allele and the *TPH2* G-703T T-allele on poorer response to treatment [15].

The three gene variants outlined above are all candidates for investigation of the genetic influence on response to CBT in anxiety disorders. However, no previous study has had sufficient sample size and power to address this in adult patients. The aim of the present study was to investigate the short- and long-term effects of the three gene polymorphisms (*5-HTT*LPR, *COMT*val158met and *TPH2* G-703T) on the outcome of CBT in a large sample of SAD patients recruited from two independent randomized controlled trials (RCTs) at two different sites. Based on previous findings on amygdala reactivity, fear extinction and CBT outcome, the hypothesis was that the s-carrier genotype of *5-HTT*LPR, the metmet genotype of the *COMT*val158met and the T-carrier type of the *TPH2* G-703T-polymorphism would be associated with reduced response to CBT, while better CBT outcome was expected for ll, val and GG carriers.

## Materials and Methods

### Design

Participants were recruited from two RCTs of CBT for SAD conducted by two independent research groups in Sweden (trial 1: n = 112 trial 2: n = 202). Participants provided symptom assessments at baseline, post-treatment and follow-up after 6 months (trial 1) or one year (trial 2). The outcomes of the respective clinical trials are reported elsewhere [34], [35]. The first trial was registered at clinicaltrial.gov (identifier NCT00564967), and the second at University Hospital Medical Information Network (http://www.umin.ac.jp/, UMIN000001383). In this study, improvement over time was tested for association with polymorphic variation in the *5-HTT*LPR, *COMT*val158met-, and the *TPH2* G-703T polymorphisms. The two trials were analyzed both together, with pooled data, and separately.

### Recruitment and Participants

The Regional Ethical Review Boards in Stockholm, Sweden, and Uppsala, Sweden, approved the study protocols, and written informed consent was obtained from all participants. Recruitment of participants was through advertising in a large Swedish newspaper, information via posters in different public places (e.g Universities and health care units), and a research web page (www.studie.nu). Consistent with the intention-to-treat principle, all participants, irrespective of the number of modules completed, were asked to complete ratings at post treatment and at follow-up. Participant characteristics are presented in Table 1. In the pooled sample both trial 1 and trial 2 were included in the analysis.

**Table 1:** Demographic variables in the two trials, listed separately and pooled together.

		*5-HTT*LPR	*5-HTT*LPR	*COMT*val158met	*COMT*val158met	*TPH2* G-703T	*TPH2* G703T
		ll	ss/sl	metmet	valmet/valval	GG	TT/TG
TRIAL 1							
**Gender**	Women n (%)	54 (72.0)	21 (28.0)	16 (21.3)	59 (78.7)	43 (62.0)	28 (38.0)
	Men n (%)	37 (86.1)	6 (14.0)	11 (25.6)	32 (74.4)	28 (66.7)	14 (33.3)
**Age**	Mean age (SD)	34 (12.0)	37 (9.6)	37 (13.3)	34 (10.7)	36 (11.5)	34 (11.5)
	Min-max	19–59	18–64	20–63	18–64	20–64	18–62
**LSAS-SR**	Pre LSAS-SR mean	66.8	71.1	70.8	66.2	68.0	72.2
**ICBT**	n (%)	17 (48.6)	42 (50.6)	16 (59.3)	43 (47.3)	35 (48.6)	22 (53.7)
**CBGT**	n (%)	18 (51.4)	41 (49.4)	11 (40.7)	48 (52.7)	37 (51.4)	19 (46.3)
TRIAL 2							
**Gender**	Women n (%)	22 (27.8)	57 (72.2)	20 (60.8)	58 (39.2)	44 (54.5)	35 (45.5)
	Men n (%)	42 (34.1)	81 (65.9)	31 (60.8)	90 (39.2)	81 (64.8)	42 (34.4)
**Age**	Mean age (SD)	37 (10.6)	38 (11.2)	39 (11.7)	38 (10.8)	37 (11.2)	39 (10.9)
	Min-max	20–63	19–71	21–66	19–71	19–71	20–66
**LSAS-SR**	Pre LSAS-SR mean	67.8	67.6	67.6	67.4	66.5	69.5
POOLED TRIALS (1+2)							
**Gender**	Women n (%)	76 (49.4)	78 (50.6)	36 (23.5)	117 (76.5)	88 (58.7)	62 (41.3)
	Men n (%)	79 (47.6)	68 (52.4)	42 (25.6)	122 (74.4)	109 (66.1)	56 (33.9)
**Age**	Mean age (SD)	37 (10.4)	37 (11.7)	38 (12.45)	36 (10.9)	37 (11.3)	37 (11.2)
	Mean-Max	19–71	18–71	20–66	18–71	19–71	18–66
**LSAS-SR**	Pre LSAS-SR mean	67.6	68.9	69.1	66.8	67.2	70.5

Legend Table 1:

LSAS-SR: Liebowitz Social Anxiety Scale – Self-Rated, ICBT: internet-delivered cognitive behavior therapy, CBGT: cognitive behavioural therapy, 5-HTT: *serotonin transporter gene, COMT: catechol-o-methyltransferase, TPH2: tryptophan hydroxylase 2.*

#### Trial 1

Participants diagnosed with SAD (n = 126) were randomized to Internet-delivered CBT (ICBT) or to cognitive behavioral group therapy (CBGT). The RCT was conducted between 2007 and 2009 at Karolinska University Hospital, Stockholm, Sweden. In brief, the participants were referred either by a primary care physician and psychiatrist or by self-referral. The SAD diagnosis was established by a psychiatrist through the Mini-International Neuropsychiatric Interview (M.I.N.I) [36]. The inclusion criteria were that participants with SAD as a principal diagnosis should not have been engaged in any CBT for the last four years, and if they received psychopharmacological treatment, they should have been stable in dosage two months prior to the study. Of 126 participants, 118 agreed to leave blood samples for DNA-analysis and 115 of these samples were successfully genotyped. Three people lacked outcome data and were not included in the analysis, which rendered 112 genotyped participants in trial 1. In the original RCT (n = 126), 64 received ICBT and 62 CBGT. Follow-up data were collected six months after treatment termination. Sixteen patients in the ICBT group and 15 in the CBGT were on stable SSRI or serotonin-norepinephrine reuptake inhibitor (SNRI) treatment during the trial. A detailed description of the original study is available elsewhere [34].

#### Trial 2

Briefly, participants diagnosed with SAD (n = 204) were randomized to ICBT or to a waitlist control group that received delayed treatment after 9 weeks. The inclusion criteria were equivalent to trial 1 (ongoing stable psychotropic medication in the ICBT group n = 10 and in the control group n = 18). Oragene saliva samples (www.dnagenotek.com) were obtained by mail from 202 participants and an independent laboratory determined the genotypes. A detailed description of the RCT is described elsewhere [35].

### Cognitive Behavior Therapy (CBT)

Internet-delivered CBT is comprised of the same components as conventional CBT, but is delivered as an online bibliotherapy, with therapist contact through encrypted email [37]. The content of ICBT was the same in both trials and based on a previously evaluated treatment manual [38] stressing the role of avoidance, negative automatic thoughts, and maintaining factors of social anxiety. As ICBT was designed as an individual treatment, the protocol was adapted from the individual CBT developed by Clark and colleagues [39]: the group protocol in trial 1 was based on the Heimberg [40] group therapy for SAD.

#### CBT in trial 1

Participants in both the ICBT and CBGT groups received therapy for fifteen weeks. All therapy was delivered by licensed clinical psychologists trained in CBT. The proportion of exposure was equal in both groups. None of the participants in the ICBT group met their therapist face-to-face, but engaged in mail contact at least once a week: participants were offered telephone or email support on demand [34].

#### CBT in trial 2

Participants in the ICBT group received nine weeks of treatment delivered by either CBT-trained psychologists or clinical psychology students under supervision of a licensed psychologist [35]. The content and the structure of the treatment was the same as in trial 1, and none of the participants met their therapist face-to-face.

### Clinical outcome measure

The primary outcome measure in both trials was the Liebowitz Social Anxiety Scale-Self-Rated (LSAS-SR) [41], [42]. The patient rated fear and avoidance on a Likert-type scale (ranging from 0– no fear/never avoid to 3– severe fear/usually avoid) for either performance situations or social interaction situations. In both trials, participants provided LSAS-SR data at pre and post-treatment and at follow-up. Trial 2 provided additional data on weekly treatment gains.

### Genotyping

DNA was extracted with standard methods from whole blood of 124 participants in trial 1 [43], and by Oragen Purifier from saliva of 202 participants in trial 2. *COMT*val158met (G472A, rs4680) and *TPH2* G-703T (rs4570625) were genotyped with TaqMan SNP genotyping assays and an ABI 7900 HT instrument (Applied Biosystems (ABI), Foster City, CA, USA) under standard conditions. The two fragments of the biallelic *5-HTT*LPR, the 336 (short) and the 379 bp (long) fragments were amplified by polymerase chain reaction (PCR) and separated by agarose gel electrophoresis. All genotypes were dichotomized; the *COMT*val158met was grouped into a val-carrier (valval, valmet) or non-carrier (metmet) genotype; the *5-HTT*LPR genotypes were grouped as either an s-carrier (ss, sl) or non-carrier (ll) genotype; the *TPH2* G-703T promoter SNP rs4570625 genotypes were dichotomized into T-carrier (TT, GT) or non-carrier (GG) groups. The allele- or genotype frequencies did not deviate from previously reported distributions in general populations [12], [44], [45]. The three SNPs were also analyzed as three genotype categories; ss, sl, ll for the *5-HTT*LPR; valval, valmet and metmet for the the *COMT*val158met and TT, GT and GG for the *TPH2* G-703T. All samples were genotyped in duplicate and genotype assessors were blind to allocation and symptom status. The distributions of all genotypes in the sample are presented in Table 1.

### Statistical analysis

Hardy-Weinberg equilibrium was verified with a chi-square test. In order to determine potential covariates on a putative association between genotype and treatment outcome, previously reported determinants were investigated for any association with genotype with an unpaired t-test normally distributed variables or Mann-Whitney U-test for non-parametric variables. None of the determinants in the RCT [34] or effect of treatment type in trial 1 were associated with genotype (p<0.05), and were not used as co-variates in further analyses (data not presented). The effects of genotype on treatment response were analyzed by repeated measure analysis of variance (ANOVA), and linear trend analysis based on week-to-week measure points in trial 2. The significance level was set at p<0.05 and statistical analyses were with SPSS 20.0.0 (SPSS. Inc., Chicago, USA) and Statview (SAS inc., Cary, USA) version 5.0.

## Results

Demographic and clinical data for the participants, grouped by genotype, are presented in Table 1. There were no significant main effects of any of the genetic variations on age, gender, treatment type or symptom severity measured with LSAS-SR at baseline, in trial 1, trial 2, or in the pooled sample.

### Effect of genotype on treatment outcome

#### Trial 1

**Figure 1:**
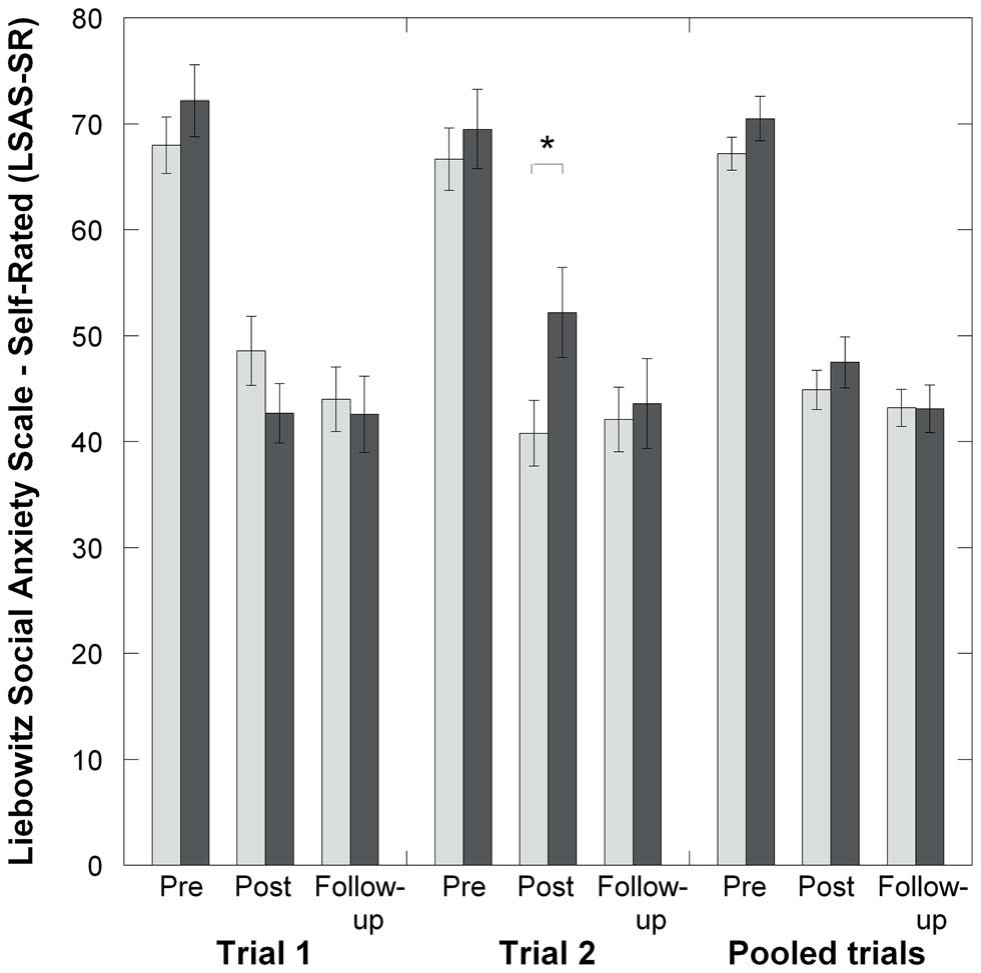
Liebowitz Social Anxiety Scale self-rated score (LSAS-SR) by *TPH2* G-703T genotype before and after cognitive behavior therapy (CBT) in social anxiety disorder (SAD) patients. Mean LSAS-SR score in G homozygotes (light grey bars) and T carriers (dark grey bars).

An interaction effect of *TPH2* G-703T genotype and time on the LSAS-SR was observed from baseline to post-treatment (Table 2. Figure 1), indicating better improvement for T-allele carriers. However, this interaction effect was not sustained at 6-month follow-up (Table 2, Figure 1). The *COMT* and *5-HTT*LPR polymorphisms did not influence CBT outcome at any point and gene-gene interactions were not observed.

**Table 2:** Pre- to post-treatment ANOVA interaction effects of the genotypes in the separated trials.

	*5-HTT*LPR	*5-HTT*LPR	*COMT*val158met	*COMT*val158met	*TPH2* G-703T	*TPH2* G703T
	ll	ss/sl	metmet	valmet/valval	GG	TT/TG
TRIAL 1						
**LSAS-SR Pre to post**	F = 0.86, df = 1,115, p = 0.36, η^2^ = 0.002		F = 0.20, df = 1,115, p = 0.65, η^2^ = 0.003		F = 4.79, df = 1,110, p = 0.03, η^2^ = 0.042	
**Pre mean (95% CI)**	67.26 (60.08–74.43)	70.96 (65.88–76.05)	67.33 (57.14–77.53)	70.62 (66.11–75.12)	68.36(63.03–73.69)	72.27 (65.41–79.13)
**Post mean (95% CI)**	47.20 (39.93–54.47)	46.54 (40.80–52.27)	42.56 (32.69–52.42)	47.99 (42.84–53.13)	48.67(42.19–55.15)	42.70 (37.01–48.39)
TRIAL 2						
**LSAS-SR Pre to post**	F = 0.46, df = 1,99, p = 0.50, η^2^ = 0.005		F = 2.93, df = 1,99, p = 0.09, η^2^ = 0.029		F = 4.42, df = 1,99, p = 0.04, η^2^ = 0.037	
**Pre mean (95% CI)**	67.17 (61.57–72.78)	70.87 (62.64–79.18)	69.37 (60.37–78.36)	67.74 (62.31–73.17)	66.79 (60.73–72.85)	70.54 (63.34–77.70)
**Post mean (95%, CI)**	46.15 (39.08–51.49)	45.29 (61.56–72.78)	41.14 (31.24–51.05)	46.91 (40.93–52.90)	40.86 (34.31–47.41)	52.16 (44.40–59.93)

LSAS-SR: Liebowitz Social Anxiety Scale – Self-Rated, 5-HTT: *serotonin transporter gene, COMT: catechol-o-methyltransferase, TPH2: tryptophan hydroxylase 2*.

#### Trial 2

A linear trend interaction effect (Table 2, Figure 1) supported better improvement over the initial 9 weeks (pre to post treatment) in the *TPH2* GG-group relative to the T-carrier group. The effect was not sustained at one-year follow-up (Table 2, Figure 1). As in trial 1, the *COMT* and *5-HTT*LPR polymorphisms did not influence CBT outcome at any point and gene-gene interactions were not observed.

#### Pooled data set

In the pooled analyses of trials 1 and 2 (n = 314), there were no significant effects of any genotype on CBT outcome (genotype by time interactions), neither at pre treatment, post treatment nor at long-term follow-up after 6 or 12 months (Table 2, Table 3). There was no evidence of gene × gene interaction effects on treatment response at any assessment point (F<1.8, p>0.18). Likewise, analysis of the SNPs as three genotype categories failed to detect any association between genotype and CBT outcome (p<0.05).

**Table 3:** Pre-treatment to follow-up ANOVA interaction effects of the genotypes reported in the separated and pooled trials.

	*5-HTT*LPR	*5-HTT*LPR	*COMT*val158met met	*COMT*val158met	*TPH2* G-703T	*TPH2* G703T
	ll	ss/sl	metmet	valmet/valval	GG	TT/TG
TRIAL 1						
**LSAS-SR Pre to** **6 month follow-up**	F = 0.21, df = 1,112, p = 0.65, η^2^ = 0.007		F = 0.32, df = 1,112, p = 0.58, η^2^ = 0.003		F = 0.94, df = 1,107, p = 0.33), η^2^ = 0.009	
**Pre mean** **(95% CI)**	67.25 (59.80–74.70)	72.80 (67.04–76.96)	70.48 (61.62–79.34)	70.56 (65.86–75.25)	69.56 (64.30–74.82)	72.07 (65.16–78.98)
**Follow-up mean** **(95% CI)**	42.34 (33.98–50.69)	44.98 (39.42–50.54)	46.36 (36.47–56.24)	43.56 (38.32–48.80)	44.43 (38.56–50.30)	42.57 (34.87–50.27)
TRIAL 2						
**LSAS-SR Pre to** **1-year follow-up**	F = 0.044, df = 1,99, p = 0.83, η^2^ = 0.00		F = 2.59, df = 1,99, p = 0.11, η^2^ = 0.003		F = 0.24, df = 1,99, p = 0.63, η^2^ = 0.004	
**Pre mean** **(95%, CI)**	67.82 (62.27–73.37)	67.59 (63.81–73.80)	67.56 (61.33–73.80)	67.41 (63.75–71.07)	66.52 (62.56–70.48)	69.53 (64.48–74.58)
**Follow-up mean** **(95% CI)**	42.39 (36.37–48.40)	42.84 (38.74–46.93)	38.58 (31.93–45.24)	44.00 (40.09–47.90)	42.12 (37.82–46.42)	43.62 (38.14–49.10)
POOLED TRIALS (1+2)						
**LSAS-SR Pre** **to follow-up**	F = 0.003, df = 1.31, p = 0.95, 0.00		F = 0.88, df = 1,314, p = 0.35, η^2^ = 0.003		F = 0.77, df = 1.314, p = 0.38, η^2^ = 0.002	
**Pre mean** **(95% CI)**	67.63 (63.19–72.05)	69.19 (66.20–72.19)	68.52 (65.72–71.46)	68.59 (65.72–71.46)	67.60 (64.45–70.75)	70.40 (66.34-74.45)
**Follow-up** **mean (95% CI)**	42.31 (37.47–47.17)	43.73 (40.44–47.02)	43.91 (40.78–47.04)	43.91 (39.45–46.37)	43.20 (39.45–46.37)	43.79 (39.02–47.93)

LSAS-SR: Liebowitz Social Anxiety Scale – Self-Rated, 5-HTT: *serotonin transporter gene, COMT: catechol-o-methyltransferase, TPH2: tryptophan hydroxylase 2*.

## Discussion

This study tested the association of three monoamine-related gene variants and response to CBT in a large sample (n = 314) of SAD patients with two independent sets of RCT data. None of the studied genetic polymorphisms in the *5-HTT*LPR, the *COMT*val158met, or the *TPH2* G-703T, was associated with long-term effect of CBT for SAD. Furthermore, no gene × gene interaction effects on the response to CBT in the pooled trials were found for the three polymorphisms. However, the G-703T polymorphism of the *TPH2* gene had a mixed short-term effect on treatment outcome in the two separate trials, the T-variant being associated with better short-term outcome in trial 1, but poorer outcome in trial 2. However, the short-time effects were not sustained at follow-up in either trial.

The site-specific results at post-treatment might be due to the minor, but potentially important, differences between the two sites, such as the duration of treatment, time to post-treatment and routines associated with the treatment procedure. Alternatively, the differences between the two cohorts could be related to unknown factors, for example a differential distribution of other relevant (unmeasured) gene variants interacting with *TPH2*. In trial 1, participants received a 15-week-long treatment in comparison with only 9-weeks of treatment in trial 1, although the outcome appeared to be equally good. It is possible the G-allelic variant of the *TPH2* G-703T had an initial effect on treatment response due to expectancy, or a placebo-like effect, which would support a previous neuroimaging study of SAD linking the anxiolytic placebo response to the G-variant of the *TPH2* G-703T [10]. In trial 1, where the treatment period was longer, the T-allele carriers had a stronger CBT response, but this effect could have occurred later in treatment, i.e. after the initial 9 weeks, when the placebo effect might be less pronounced. However, this interpretation is tentative and needs further investigation.

The current results are not entirely consistent with some previous therapygenetic studies that have found association between *5-HTT*LPR and response to CBT in a broader set of diagnoses after six months or longer post-treatment [5], [14]. These studies failed to demonstrate an effect of genotype immediate after treatment but at follow-up, whereas another study on panic patients, reported a significant effect of genotype both pre and post treatment [24]. The lack of replication in the current study could be due to notable differences between the studies regarding age and diagnosis (e.g. children with any anxiety-related problems [5], [14] in comparison to adults with only SAD in the present study). In contrast to the study on panic disorder by Lonsdorf and colleagues [13], an effect of the *COMT*val158met polymorphism on CBT outcome could not be demonstrated. It is not known how specific diagnoses affect different candidate genes for therapeutic responsiveness, that is, whether the same genes are relevant for CBT response across disorders.

The preliminary null findings previously reported by Hedman et al [3], regarding genetic influences of *COMT*val158met and *5-HTT*LPR on CBT outcome in SAD remained null findings in the present study even with a substantially increased sample size, and there was no influence of *TPH2* G-703T polymorphism on long-term treatment response even with pooled data. Thus, none of these polymorphisms, either separately or interactively, had robust effects on the long-term response to CBT for SAD. It should also be noted that none of the polymorphisms were associated with symptom severity (LSAS-SR) before treatment.

Despite a strict treatment manual, there were differences between the sites and some general limitations to the study. The sample size was too limited to reveal small genetic effects, and for the *TPH2* G-703T only a few participants had the TT genotype. With a larger sample, gene × gene interactions could be investigated more exhaustively. Besides differences in the duration of treatment between the sites, there were disparities in parts of the procedure, for instance, the amount of contact the participant had with the caregiver. In trial 1, the participants met their psychiatrist for screening at the beginning of the treatment, whereas, in trial 2, screening was via telephone and online. In addition, in trial 1 half of the group was randomized to CBGT, whereas, in trial 2, all participants received ICBT. Although the site-specific disparateness could account for differences in the outcomes in the study, it is reasonable to assume that strong genetic effects would manifest despite these differences. Psychotherapy varies greatly in a face-to-face setting, however, ICBT is highly standardized and adherence to the treatment protocol is monitored through the treatment format, that is, all communication between therapist and patient is stored.

The important strengths of the study were the standardized and roughly equivalent treatment protocols and outcome measures across both trials and in particular the large sample size, which could be crucial in therapygenetics. Large-scale replication studies at different and independent sites would demand similar treatments in both the content of the therapy and the evaluation of the results. In this study, the treatment was standardized and the outcome measure was identical.

In conclusion, none of the *5-HTT*LPR, *COMT*val158met and *TPH2* G-703T-polymorphisms, either separately or interactively, affected the long-term response to CBT for SAD. Therefore, there is a need for larger and better cohorts evaluated with standard outcome measures, as this might be essential for identifying genetic predictors of response to psychological treatment.
