# Correction: Genetic Polymorphisms in Monoamine Systems and Outcome of Cognitive Behavior Therapy for Social Anxiety Disorder

**DOI:** 10.1371/journal.pone.0165249

**Published:** 2016-10-18

**Authors:** Evelyn Andersson, Christian Rück, Catharina Lavebratt, Erik Hedman, Martin Schalling, Nils Lindefors, Elias Eriksson, Per Carlbring, Gerhard Andersson, Tomas Furmark

In the abstract, the trial number is incorrect. Please see the corrected trial number here: NCT00564967.
